# PES Surface Modification Using Green Chemistry: New Generation of Antifouling Membranes

**DOI:** 10.3390/membranes6020023

**Published:** 2016-04-18

**Authors:** Norhan Nady

**Affiliations:** Polymeric Research Department, Advanced Technology and New Materials Research Institute, City of Scientific Research and Technological Applications (SRTA-City), New Boarg El-Arab City 21934, Alexandria, Egypt; Norhan.Nady77@yahoo.com; Tel.: +20-109-091-8521

**Keywords:** enzyme-catalyzed modification, poly(ethersulfone) membrane, protein repellence, antifouling membranes, ferulic acid, laccase, green chemistry

## Abstract

A major limitation in using membrane-based separation processes is the loss of performance due to membrane fouling. This drawback can be addressed thanks to surface modification treatments. A new and promising surface modification using green chemistry has been recently investigated. This modification is carried out at room temperature and in aqueous medium using green catalyst (enzyme) and nontoxic modifier, which can be safely labelled “green surface modification”. This modification can be considered as a nucleus of new generation of antifouling membranes and surfaces. In the current research, ferulic acid modifier and laccase bio-catalyst were used to make poly(ethersulfone) (PES) membrane less vulnerable to protein adsorption. The blank and modified PES membranes are evaluated based on e.g., their flux and protein repellence. Both the blank and the modified PES membranes (or laminated PES on silicon dioxide surface) are characterized using many techniques e.g., SEM, EDX, XPS and SPM, *etc.* The pure water flux of the most modified membranes was reduced by 10% on average relative to the blank membrane, and around a 94% reduction in protein adsorption was determined. In the conclusions section, a comparison between three modifiers—ferulic acid, and two other previously used modifiers (4-hydroxybenzoic acid and gallic acid)—is presented.

## 1. Introduction

The interaction between a material and its environment takes place at its surface. Therefore, surface properties often dictate the performance of the material and modifying of the surface leads to the production of novel materials or materials with novel properties [1,2]. The surface functionalization of (polymeric) membranes has already become a magic key in membrane manufacturing. The intention of surface modification of membranes is either to minimize unwanted interactions (adsorption/adhesion) which reduce the performance (membrane fouling), or to introduce additional (tailored) interactions (affinity, responsiveness, or catalytic properties) for improving the selectivity or creating novel separation function [3,4].

Membrane fouling, that is defined as the accumulation of substances on the membrane surface and/or within the membrane pores which results in deterioration of membrane performance during operation, is the major obstacle in all kinds of membrane processes. There are many types of fouling which depends on the type of foulant itself (*i.e.*, the component that can adsorb/accumulate on membrane surface or inside the membrane pores and causes fouling). The types of fouling are classified into five main types: colloidal fouling [5], scaling fouling [6], antifoam fouling [7], protein fouling [8,9], and membrane biofouling [10,11]. These types of fouling can be reduced using hydrodynamic [12], surface modification [13], and regular cleaning methods [14]. From a practical point of view, membrane fouling is a more complicated phenomenon that cannot be solved individually because the formed fouling layer most probably consists of more than only one foulant type. Once a type of foulant is attached or adsorbed to the membrane surface, it works as an initiator for attachments of other types of foulants. For example, protein adsorption or scaling fouling by sulfate salts can be a primary step for attachment and growth of live cells (*i.e.*, biofouling formation) [15,16].

Membrane material has a strong influence on membrane fouling; *i.e.*, the type of material determines the physicochemical interactions between the membrane and the substances in feed solution. Both membrane surface hydrophilicity and membrane surface structure are the main tools to mitigate protein fouling and consequently biofouling [17]. Protein repellence depends on the fact that the hydrophilic surface attracts so much water that adsorption of proteins is reduced [18]. On the other hand, the surface structure has significant impact on membrane antifouling performance by keeping the foulant (protein molecules) at a distance from the surface (steric hindrance) that reduces intimate contact between the foulant and the surface [1,3,17,18]. As both hydrophilicity and surface structure of the membranes are important, membrane manufacturers have tried to graft polymers of different kinds either on the ready-made membrane [19] or on the polymer from which the membrane is prepared [20]. This grafting has mostly been initiated with a glow discharge apparatus, electron beam-induced, plasma, or UV irradiation [13,20,21].

**Figure 1:**
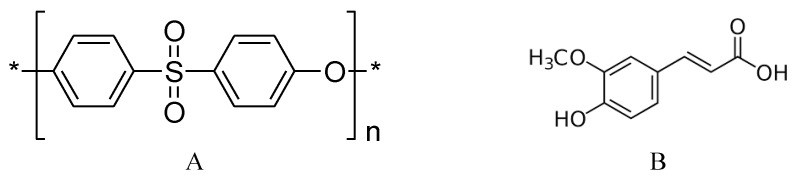
Molecular structure of (**A**) PES and (**B**) ferulic acid.

**Figure 2:**
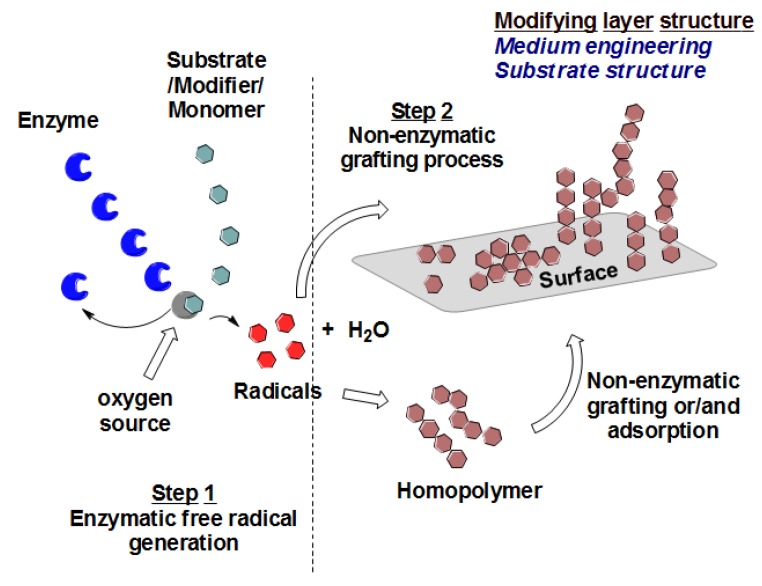
Schematic representation of enzyme-catalyzed modification of PES surfaces.

Polyethersulfone (PES, see Figure 1) is the thermoplastic material of choice for the manufacture of various types of membranes. This material increases the robustness of membranes due to its structural and chemical stability. Unfortunately, PES is a hydrophobic material, with a relatively low surface energy and high water contact angle, and membranes made from such material are more vulnerable to adsorptive fouling. In order to capitalize on the usefulness of PES membranes in separation processes, surface modification of this material to make it more polar and less hydrophobic was at the core of many studies [22,23]. Excellent results have been achieved by using different techniques such as blending [24,25] and photoinduced grafting [20,26]. Recently, enzymatic-grafting surface modification of PES membrane was investigated [27]. In this modification, as shown in Figure 2, enzyme laccase from *Trametes versicolor* is used to oxidize 4-hydroxybenzoic acid and gallic acid to their corresponding free radicals that are subsequently grafted onto PES membranes (surfaces), introducing polar groups (OH, COOH) on the membrane surface mainly through O-centered coupling. Other monomers can be oxidatively grafted onto the attached monomers, to form oligomers or polymers, which may lead to additional C–O as well as C–C bond formation with concomitant coloration of the surfaces [28]. Also, grafting and/or strong adsorption of the formed homopolymers formed in the reaction medium can be done. Both of the modification condition and the structure of the modifier itself can be used to change the shape/structure of the formed modifying layer.

This novel surface modification of PES membrane that uses green chemistry to graft polymeric oligomers on the PES membrane surface will provide for a wide research criterion in the future to create a new generation of anti-fouling membrane materials.

Ferulic acid (Figure 1) is a naturally occurring phenolic acid widely used in cosmetics, food, and pharmaceutical industries owing to its effective antioxidant, antimicrobial, and anticancer activities [29,30]. A laccase from *Trametes hirsuta* was used to coat flax fibers and fabrics with hydroquinone and various methoxyphenols to obtain antibacterial surfaces; the combination of ferulic acid and hydroquinone resulted in a coating with the best antibacterial performance against *Bacillus substilis* and *Staphylococcs aureus* [31,32]. The antibacterial effect of ferulic acid makes it a promising candidate to add (graft) an antibacterial layer onto the PES layer to obtain a novel copolymer of ferulic acid and PES surface. Ferulic acid was proposed as a successful modifier for PES surfaces [27], and its ability to form a modifying layer to reduce attachment of *Listeria monocytogenes* pathogenic bacterium [33] has been reported. The modification of the PES surface using this modifier still needs further investigation from the point of view of membrane performance, which will be presented in this paper, as well as the structure of the formed poly(ferulic acid) on PES surface, which is currently being examined in our lab.

This article provides a new modified layer of poly(ferulic acid) on the PES membrane using the enzyme-catalyzed modification (grafting) technique. An evaluation of the performance of the modified membranes from the standpoints of flux, grafting yield, and protein adsorption was presented. These performances are related to the various modification parameters. These parameters include modification time, modifier concentration, laccase concentration, modification temperature, modification pH and type and strength of the used buffer. SEM images aid in explaining the performance of different modified real membranes. Furthermore, EDX and XPS are used in analysis of the modified real membranes. Moreover, model PES surfaces (described in method section) are used to determine the thickness of the formed poly(ferulic acid) layer using ellipsometry, and the shape of the formed layer was imaged by SPM. Also, the effect of modification on the static water contact angle was investigated using the model surface. A comparison between the obtained results using ferulic acid and previously used modifiers [17,27,28] (*i.e.*, 4-hydroxybenzoic acid and gallic acid) is presented in the conclusions section.

## 2. Results and Discussion

In previous research [27], we have found that PES membranes can be modified with phenolic acids by the action of laccases. Experimental results indicated that the modified PES membranes using both 4-hydoxybenzoic acid and gallic acid possess favorable properties. Here, we study the performance of the modified membranes using ferulic acid modifier. We will first discuss the chemical analysis of both blank and modified real membranes. Then, the obtained results regarding membrane color change, grafting yield, BSA adsorption, together with SEM images of PES real membranes modified with ferulic acid under different modification conditions will be presented and discussed. The water contact angles on model PES surfaces on silicon dioxide slides were determined. Moreover, SPM was used to image both blank and modified model PES surfaces. In the last section, an outlook is given for enzyme-catalyzed modification using the three phenolic modifiers (ferulic acid and previously studied 4-hydroxtybenzoic acid and gallic acid).

XPS analysis was carried out for both blank and modified real membranes and the results are shown in Table 1. The obtained results show a decrease in sulfur content (about a 65% reduction), as observed in the intensity of the S*_2p_* peak at 169.0 eV (–SO_2_–). This decrease can be considered as an indication of the formation of an overlayer on the membrane that covers the underlying sulfur. The concentrations of both carbon and oxygen are notably different from the blank membrane, which is another indication of the formation of an extra layer. The presence of C=O peaks and the nitrogen in the blank membrane are most probably due to the presence of other used additive materials like polyvinylpyrrolidone as well as due to incomplete leaching out of used solvent during the phase-inversion fabrication of the PES membrane. The decrease in the C=O peak upon the covalent coupling of ferulic molecules can be returned to the decarboxylation as illustrated in previous research [27].

**Figure 3:**
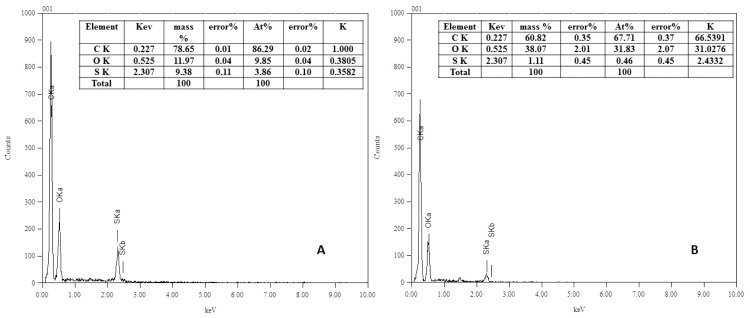
EDX analysis spectra of (**A**) Blank and (**B**) Modified real membrane using 10 mM ferulic acid, 0.5 U∙mL^−1^ laccase, 45 °C, pH 5 (0.1 M sodium acetate buffer) and 2 h modification time.

EDX analysis (see Figure 3) was carried out for the blank and the modified real membranes using ferulic acid (10 mM modifier, 45 °C, pH 5 (0.1 M sodium acetate buffer) and 2 h modification time). In this analysis, X-rays are generated in a region greater than one micron in depth, which enables structural analysis more deeply inside the membrane.

The EDX results are in good agreement with XPS results. The sulfur content decreased upon modification from 9.38 mass% for the blank to 1.11 mass% for the modified membrane (atomic%: 3.86 for the blank and 0.46 for the modified membrane). This decrease in sulfur mass% of the modified membranes supports the XPS indication of formation of poly(ferulic acid) layer on the surface of the blank membrane. Regarding the oxygen, the mass% increased from 11.97 to 38.07. This is extra evidence of the addition of oxygen molecules with modification. The absence of nitrogen is due to the use of a detector that detects the elements from sodium and does not detect the nitrogen element. Also, the X-rays are generated at depths reaching 1000 nm, whereas XPS rays generate on the surface at around 5 nm. The polyvinylpyrrolidone additive may be concentrated very near to or on the membrane surface, which may be detected by XPS and it is difficult to measure by EDX. The overall benefit of EDX is supporting the XPS results showing the formation of a new layer on the real PES membrane.

**Figure 4:**
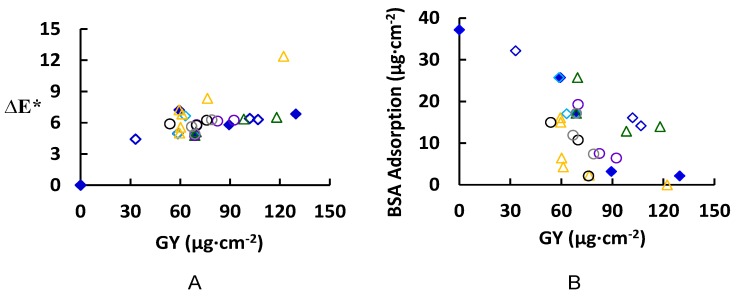
Membrane color change (∆E*) (**A**) and BSA adsorption (total adsorption equals reversible + irreversible adsorbed protein) (**B**) with grafting yield (GY); the common reaction condition is 4.8 mM ferulic acid, 0.5 U·mL^−1^ laccase, 2 h modification time, 25 °C, pH 5, and 0.1 M sodium acetate buffer. The following parameters were studied: modification time (0.5, 2, 8, and 24 h) with 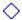 0.6 mM ferulic acid, and 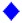 4.8 mM ferulic acid, 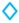 ferulic acid concentration (0.6, 1.2, and 4.8 mM) at 8 h modification time, reaction temperature (25, 35, 45, 55, 65 and 75 °C) at 0.5 h modification time, 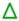 reaction pH (4, 5, 6, and 7), 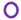 enzyme concentration (0.25, 0.5, 0.75, and 1 U·mL^−1^), 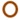 buffer type (sodium citrate, citrate phosphate, sodium acetate, and phosphate), 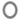 buffer strength (0.05, 0.1, and 0.5 M). Typical errors: ± 0.2 for membrane color change (∆E*) and ± 0.3 µg∙cm^−2^ for BSA adsorption.

The grafting yield (GY) was determined together with the membrane color change (∆E*), and the amount of total BSA that adsorbs (reversible and irreversible) to the surface are illustrated in Figure 4 for various modification conditions. The reader should keep in mind the presence of two competitive reactions; the bonding of ferulic acid on the PES surface and the bonding of the ferulic molecules with each other inside the solution (homopolymers formation). The change of membrane color (∆E*, see Figure 4A) increased with increasing GY, up to 60 µg∙cm^−2^. At much higher GY, the total change in color started to level off except for at high modification temperatures (higher than 45 °C). This behavior may be explained by the formation of dense layers that are more saturated in color. Color saturation (not measured in this work) is a characteristic indicating the vibrancy or intensity of a color; color with high saturation will appear more intense than the same color with less saturation. At higher temperature, the overall reaction rate increases with a large increase in GY (61.11 µg∙cm^−2^ at 55 °C to 76.4 µg∙cm^−2^ at 65 °C) [34]. The increase in the GY corresponds to the increase in the modifying layer thickness (on model surface) from 11.5 ± 0.05 nm to 13.6 ± 0.4 nm. The increases in GY with very slight change in thickness can be a result of an increase in the grafting density (grafted chains/oligomers per unit area) and color saturation of the surface that reflated on increase in the membrane color change. However, the contact angle at both reaction temperatures does not change (Blank model surface: 78.8° ± 1°, modified model surface at 55 °C and 65 °C are 63.4° ± 1° and 64.4° ± 0.3°, respectively). On the other hand, the difference in total protein adsorption on real membranes at both temperatures is not significant, however, the reduction in the total protein adsorption relative to the blank real membrane is remarkable as shown in Figure 4B (total protein adsorption for blank real membrane is 37.18 µg·cm^−2^ and for modified membranes at 55 °C and 65 °C are 4.29 µg∙cm^−2^ and 2.25 µg∙cm^−2^, respectively). The pH has a pronounced effect on the GY: for example, at pH 5 the GY after 2 h modification is 68.76 µg·cm^−2^ (17.15 µg·cm^−2^ total protein adsorption), while at pH 7 it is 118 µg·cm^−2^ (13.95 µg·cm^−2^ total protein adsorption/44.19° ± 0.6° corresponding contact angle on model surface). This is attributed to the ionization of ferulic acid at higher pH, which leads to a lower oxidation potential, and thereby a higher reaction rate and greater GY [35]. Also, laccase concentration had a significant effect on both the GY and protein adsorption. For example, an increase of the laccase concentration from 0.5 to 0.75 U∙mL^−1^ causes an increase of the GY from 68.76 to 82.4 µg·cm^−2^ and reduction of the total protein adsorption from 17.15 to 7.52 µg·cm^−2^. Using higher enzyme concentration does not have a noticeable effect on total protein adsorption although an increase of the GY to 92.33 µg·cm^−2^ has been detected. However, the buffer strength had a minor influence on the GY [36], the buffer type had a remarkable effect on the GY and, consequently, the reduction in the total protein adsorption. For example, using sodium citrate instead of sodium acetate under the same modification conditions (4.8 mM ferulic acid, 0.5 U∙mL^−1^ laccase, 25 °C, pH 5, 0.1 M buffer, 2 h modification) resulted in an increase of the GY from 68.8 to 75.9 µg∙cm^−2^, and a reduction in total protein adsorption (17.1 to 2.1 µg∙cm^−2^ for sodium acetate and sodium citrate, respectively). These results can be attributed to the effect of buffer on the activity of the laccase enzyme. To illustrate the effect of buffer type on the activity of enzyme laccase, the enzyme assay was determined using the different buffer types and the results showed that the best buffer for higher enzyme activity is in the following order: phosphate > sodium citrate > citrate phosphate > sodium acetate.

From a first look at Figure 4B, we can say that there is no straightforward relationship between the GY and the total protein adsorption except in the case of using a low concentration of ferulic acid (0.6 mM, non-solid blue diamond). There are many modified membranes that show a remarkable reduction in total protein adsorption; for example, modified real membranes under the following conditions: (1) 4.8 mM ferulic acid, 0.5 U∙mL^−1^ laccase, 25 °C, pH 5, 0.1 M sodium acetate buffer, 8 h modification time (total protein adsorption is 3.21 µg∙cm^−2^), (2) 4.8 mM ferulic acid, 0.5 U∙mL^−1^ laccase, 65 °C, pH 5, 0.1 M sodium acetate buffer, 0.5 h modification time (total protein adsorption is 2.25 µg∙cm^−2^), (3) 4.8 mM ferulic acid, 0.75 U∙mL^−1^ laccase, 25 °C, pH 5, 0.1 M sodium acetate buffer, 2 h modification time (total protein adsorption is 7.52 µg∙cm^−2^), and (4) 4.8 mM ferulic acid, 0.5 U∙mL^−1^ laccase, 25 °C, pH 5, 0.1 M sodium citrate buffer, 2 h modification (total protein adsorption is 2.2 µg∙cm^−2^).

From the above stated examples for the modified membranes that showed a significant reduction in total protein adsorption, most remarkable is the low concentration of ferulic acid in the four cases. The low concentration of ferulic acid allows for the minimization or prevention of both unwanted homopolymer formation and adsorption on the PES surface and unwanted crosslinking between the grafted oligomers [30].

**Figure 5:**
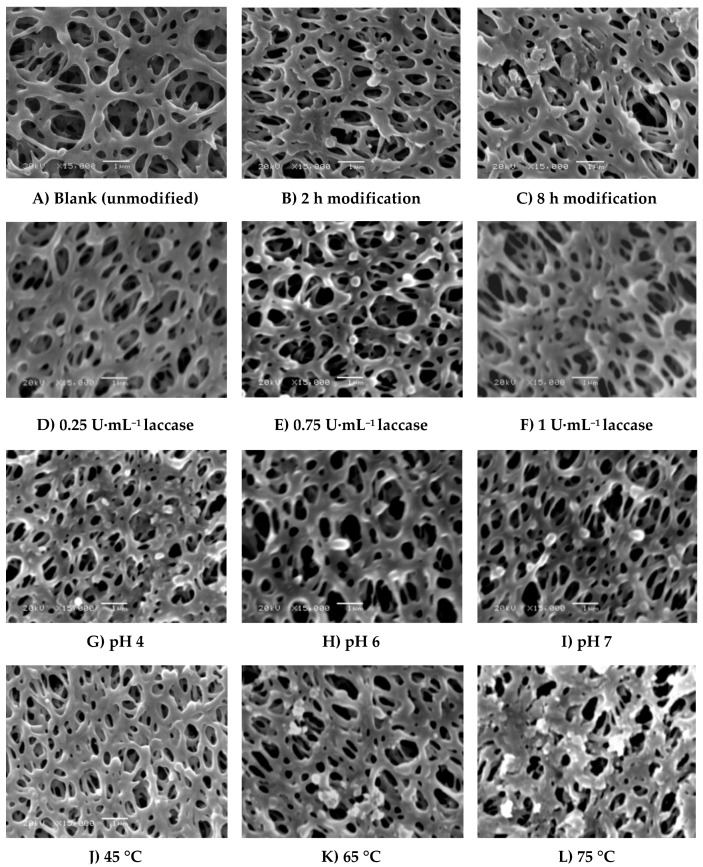
SEM photos (15,000× magnification, 1 µm bar) of real (**A**) blank membrane and modified membranes under different modification conditions. Common modification condition is (**B**) 4.8 mM ferulic acid, 0.5 U∙mL^−1^ laccase, 25 °C, pH 5, 0.1 M sodium acetate buffer, 2 h modification time. The effect of different modification conditions was illustrated as follows: (**C**) modification time (8 h); (**D**–**F**) modified membranes using 0.25, 0.75 and 1 U∙mL^−1^ laccase concentrations, (**G**–**I**) modified membranes at pH 4, 6, and 7, (**J**–**L**) modified membranes at 45 °C, 65°C, and 75°C with 0.5 h modification time.

**Figure 6:**
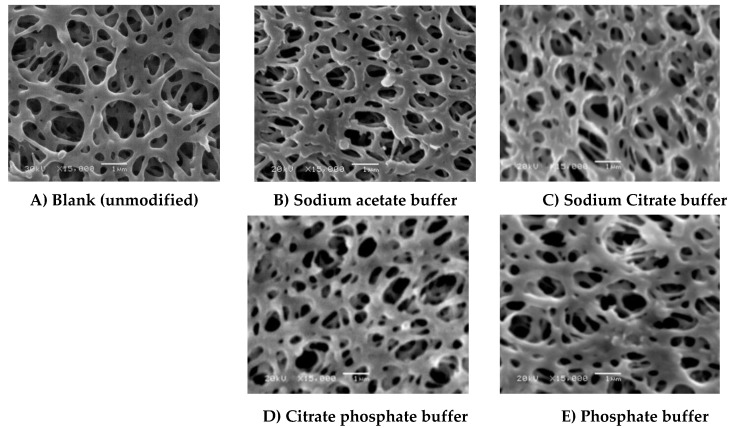
SEM photos (15,000× magnification, 1 µm bar) of (**A**) Blank membrane and (**B**–**E**) modified membranes using different buffers. Common modification condition are 4.8 mM ferulic acid, 0.5 U∙mL^−1^ laccase, 25 °C, pH 5 using 0.1 M buffer, and 2 h modification time: (**B**) Sodium acetate buffer; (**C**) Sodium citrate buffer; (**D**) citrate phosphate buffer and (**E**) phosphate buffer.

SEM photos of real blank membranes and membranes modified with ferulic acid are shown in Figure 5 and Figure 6. The following modification conditions were used as common modification parameters to investigate the change in morphology of the membranes due to change in condition: 4.8 mM ferulic acid, 0.5 U∙mL^−1^ laccase, 25 °C, pH 5, and 0.1 M sodium acetate buffer, 2 h modification time (Figure 5B and Figure 6B). As shown in Figure 5C, the thickness of the formed ferulic acid layer seems to increase with increasing modification time. Also, we can notice the formation of extend domains from the porous edges and on the lamella surface. However, the pores are still open and, consequently, the flux reduction is very slight (3.9% reduction due to irreversible protein adsorption corresponding to 24.5% reduction in flux of blank membrane). Increase of the laccase concentration (Figure 5D–F) seems to affect the GY and, consequently, the protein repellence (56.15% reduction in total protein with increase in the laccase concentration from 0.5 to 0.75 U∙mL^−1^). Increase in the enzyme concentration to 1 U·mL^−1^ resulted in increase of the GY (10 µg∙cm^−2^) with only a 1.1 µg∙cm^−1^ reduction of total absorbed protein. On the other hand, using high concentrations of the enzyme is not recommended for economic reasons.

Application of low pHs (4 and 5) (see Figure 5B,G, respectively) results in the formation of a thin layer over the membrane surface, while increasing the pH from 5 to 7 (Figure 5I) seems to result in binding of small lumps of material, most probably homopolymers. However, using low concentrations of ferulic acid (4.8 mM) resulted in the disappearance of the huge lumps for any of the chosen conditions as observed in the case of using 4-hydroxybenzoic acid shown in Figure 4 in previous research [28]. This is because the reaction of ferulic acid is somehow similar to gallic acid reaction. The reaction of both of them is very fast and the modification process seems to be initiated across the entire membrane surface at the same time and the deposited layers seem uniform except in the case of modification at high temperature as shown in Figure 5L. The protein adsorption under this condition is lower than the detection limits, and no change in the flux due to irreversible protein adsorption was determined. The change of buffer affects not only the GY but also the shape of the formed layer (see Figure 6), which can be related to the inhibition effect of buffer ions on the laccase activity as mentioned before. Sodium citrate buffer shows a different surface morphology compared to the other buffers and the lowest total protein adsorption (2.12 µg∙cm^−1^) among them under the tested modification conditions. Depending on the different morphologies obtained and the higher activity of the used enzyme using other buffers that do not contain acetate and/or sodium ions, further investigation using these buffers is required.

Table 2 shows the flux reduction due to modification, the flux reduction due to irreversible protein adsorption (the flux was determined after back and forward washing of the membranes to remove the reversible adsorbed BSA), and the total flux reduction of six selected modified real membranes and the blank one. Moreover, the GY and the total protein adsorption (reversible and irreversible) have been included. Also, the same conditions have been used to modify model PES surfaces and both the static water contact angle and the thickness of formed modifying layers were determined using ellipsometry as described in the experimental section. The reason for using model PES surface is the presence of other additives such as polyvinylpyrrolidone as illustrated by XPS analysis that affects the water contact angle. Also, the organic layer on silicon dioxide silicon support is required for thickness measurement by the ellipsometry.

**Figure 7:**
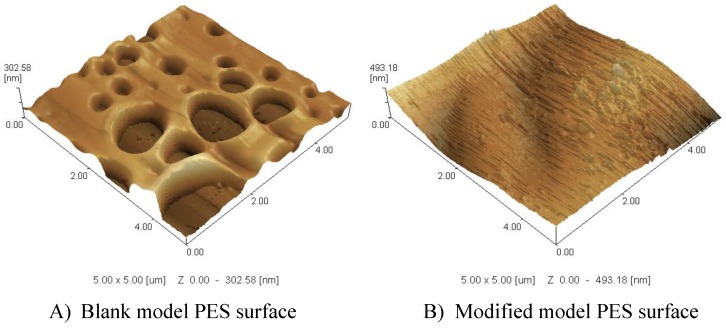
SPM 3D (5 µm × 5 µm) images of PES model surface (**A**) and modified model PES surfaces and (**B**) prepared at 10 mM ferulic acid, 0.5 U·mL^−1^ laccase, 45 °C, pH 5 (0.1 M sodium acetate buffer) and 2 h modification time.

For all reaction conditions tested, the blank real membrane shows high flux reduction (24.5%) due to irreversible protein adsorption; whereas modified membranes always showed higher residual fluxes as shown in Table 2 as examples. The modification of the different conditions resulted in reduction in total protein adsorption, however, the increase in the GY did not have a proportionate effect on the reduction of the total protein adsorption. A clear observation is that the highest GY (in this table, 118 µg∙cm^−1^) does not provide the best protein reduction, whereas it corresponds to the lowest contact angle (44.2°). This goes against the proposal that the increases in the hydrophilicity of the PES surface will result in a reduction of the adsorbed protein, but the layer structure is quite important. On the other hand, the highest GY showed the smallest layer thickness on model PES surfaces, which supports increasing the number of grafted oligomers/polymers, and not their length as illustrated in the previous section in which an increase in the color depth of the real membrane was proposed. Also, crosslinking is possible due to the presence of three legs in the structure of ferulic acid as explained in previous work [17,28]. The layer structure can be turned by the modification conditions and the choice of modifier. Modifiers with more than two reactive groups or legs (like ferulic acid) will lead to denser 3D networks as shown in Figure 7, while molecules with only two reactive groups give linear or branched structures. As illustrated in SPM, the ferulic acid forms a pancake-like structure (3D) formed on the blank PES layer. The ability of ferulic acid to form network as crosslinker is pronounced in its reaction on the membrane model surface to form an extended 3D layer. The relatively high used concentration (10 mM) of the modifier (ferulic acid) combined with the relatively high temperature (45 °C) assisted the formation of this pancake-like layer.

Another important observation is regarding the “common modification” of the membrane showing a very small flux reduction due to irreversible protein adsorption (1.4%), while the value for total protein adsorption was the highest among all tested membranes (17.1 µg∙cm^−1^). This indicates that most of the adsorbed protein on this modified real membrane is reversible and can be washed out in contrast to the case of the blank membrane. Regarding the results using the sodium citrate buffer, the identified flux reduction due to irreversible protein adsorption was high compared to others (5.6%) while the determined total protein adsorption was the lowest among the reported values in this table (2.1 µg∙cm^−1^). For that, most probably the adsorbed protein on this membrane cannot be removed, which is not desirable.

## 3. Experimental Section

### 3.1. Chemicals and Enzyme Activity

Ferulic acid (>98%), catechol (>99%), and Laccase from *Trametes versicolor* (10.4 U·mg^−1^) were obtained from Fluka. From Sigma-Aldrich, sodium acetate (anhydrous, ≥99%), sodium dihydrogen phosphate (anhydrous, ≥98%), disodium hydrogen phosphate (anhydrous, ≥98%), citric acid (99.9%), Bovine Serum Albumin (BSA, lyophilized powder, ≥96%) and acetic acid (99.9%) were purchased. Dichloromethane (DCM) was purchased from Merck. Poly(ethersulfone) (PES) polymer was obtained from BASF (Germany). Prime grade 150 mm silicon wafers with 2.5 nm native oxide layer was purchased from Wafer Net Inc (San Jose, CA, USA). Flat sheet commercial polyethersulfone membranes were purchased from Sartorius (0.2 µm pore size, flow rate of pure water is >28 mL·min^−1^·cm^2^ at ∆*P* = 1 bar). Laccase activity was carried out with catechol as substrate as described in our previous work [28]. One unit of laccase activity is determined as the amount of enzyme required to oxidize 1 µmol of catechol per min at 25 °C. The reaction time is 10 min.

### 3.2. Membrane Characterization

The CIELAB coordinates for the modified membranes were measured with a ColorFlex (HunterLab spectrophotometer, D 65/10°, Reston, VA, USA) as described in our previous work [28]. Aperture size was 8 mm diameter. The membranes were washed by filtration with at least 200 mL deionized water and then dried for 48 h before the actual color change was measured. The color values L* (lightness), a* (red-green axes), b* (yellow-blue axes), and ∆E* were determined using compare mode and the blank membrane was used as the standard surface. ∆E* is the degree of color change, which is calculated from [(L*)^2^ + (a*)^2^ + (b*)^2^]^0.5^. All parameters were determined as the average of three readings of each membrane circle.

Real blank and modified PES membranes were imaged using a Scanning Electron Microscope (SEM, Jeol Jsm 6360LA, Shimadzu, Japan). The membrane samples were cut using a very sharp shaving blade and were coated with Au, and were imaged at a voltage of 30 KV, and a resolution of 1280 × 960 pixels. Elemental analysis of the modified membranes was carried out by using SEMEDX- Integrated Analysis System. The Energy-dispersive X-ray Spectroscopy (EDX or EDS) analysis system works as an integrated feature of SEM (Si Li detector, detection area 10 mm^2^, Shimadzu, Japan)]. The X-rays are generated in a region greater than one micron in depth of real membranes. A JEOL JPS-9200 X-ray Photoelectron Spectrometer (XPS, Shimadzu, Japan) was used for surface analysis of the elemental composition of the real modified membranes to a depth of around 5 nm as described in our previous work [27]. High-resolution spectra were obtained under UHV conditions using monochromatic Al Kα X-ray radiation at 12 kV and 25 mA, using analyzer pass energy of 10 eV. All high-resolution spectra were corrected with a linear background before fitting.

A dead-end stirred ultrafiltration cell (Millipore, Model 8050, active transport area 13.4 cm^2^) was used to characterize the filtration performance of real blank and modified membranes as done in our previous work [28]. The amount of material grafted onto the membrane surface is determined from the weight of the membrane, before and after grafting, and the grafting yield is expressed as the weight increases relative to the membrane surface area. Before grafting, all the membranes were kept for 48 h in desiccators to keep them dry.

Bovine Serum Albumin (BSA) was used as a model compound to evaluate total protein adsorption on real blank and modified membranes as illustrated in our previously published paper [28]. Briefly, a standard curve using different prepared BSA concentration was prepared using spectrophotometer at 280 nm wavelength. The membrane circles were shaken for 24 h in 50 mL 1g∙L^−1^ BSA concentration and then the non-adsorbed BSA was determined. The adsorbed BSA (*i.e.*, reversible and irreversible adsorbed protein) was calculated from the difference between the initial and residual BSA concentrations relative to membrane surface area.

The membrane flux after BSA adsorption was carried out as follows. First, the membrane was backwashed using 200 mL of deionized water to remove any unbound BSA (reversible adsorbed protein), and then forward washed using 200 mL of deionized water. After that, fresh deionized water was used in forward motion at 1 bar applied pressure, and the flux was determined. The reported values are the average of three independent measurements.

Laccase-catalyzed modified model PES surfaces (see next section) were characterized by static water contact angle measurements performed using Krüss DSA 100 apparatus. A drop of demineralized water (7 µL) was deposited on three different spots of each spin-coated (model) sample and the average was calculated. Scanning Probe Microscope (SPM, Shimadzu, Japan) was used to image model PES surface prepared as described below. Non-contact mode was used to image 5 µm × 5 µm.

### 3.3. Preparation of Model Surfaces and Layer Thickness

The preparation of model surface was described in previous research [17] and will be described here briefly. The thickness of the native silicon dioxide layer (2.5 nm) was increased to approximately 70 nm by heating at 1000 °C for 100 min. Then, the wafers were cut into strips of 1 cm × 4.5 cm and were sonicated in ethanol for 15 min, washed with water and ethanol, and dried in flowing nitrogen. The thickness and refractive index of silicon dioxide layer were determined with a computer-controlled null ellipsometer (Sentech instruments Gmbh) at λ = 632.8 nm and angle of incidence 70°. Values of 3.85 and 0.02 were used for the refractive index (*n*) and the imaginary refractive index (*k*) of silicon [37,38], respectively. Then, the strips were given a plasma treatment (PDC-32G, Harrick at RF-level high) for 10 min and were used as substrate for the model PES surfaces by spin coating them with 0.25% w/w PES solution in dichloromethane for 10 seconds at 2500 rpm. Subsequently, the PES coated strips were put at 300 °C for 60 min. The thickness and refractive index of PES layers deposited on silicon dioxide were determined by ellipsometry. The spin-coated PES model surfaces were then modified using laccase (see respective section). The surfaces were kept for 24 h in glass-covered dishes in desiccators supplied with silica gel to remove any moisture. After drying, the thickness and refractive index of the modification layer was determined by ellipsometry. The refractive index initial values used for the silicon dioxide, PES, and modification layers were 1.46, 1.65, and 1.57, respectively [37]. Each measuring value was taken as an average of three different reads from three different locations on two different strips.

### 3.4. Membrane Modification Experiments

Flat membranes were modified as described in previous work [17,28]. Briefly, membranes were incubated in 40 mL 0.1 M sodium acetate buffer (pH 5) containing different concentrations of ferulic acid (monomer) and enzyme laccase. Ferulic acid, was used at concentrations of 0.6, 1.2, 4.8, 10.0, and 28.8 mM, and modification times of 0.5, 1, 2, 8, and 24 h. The enzyme concentrations tested were 0.1, 0.5, 0.7, and 1 U·mL^−1^. Different reaction temperatures (25, 35, 45, 55, 65 and 75 °C) were tested. After the modification time was completed, the membranes were washed by strong flushing with water, repeated dipping in deionized water and subsequent decantation. The modified membranes were kept in glass-covered dishes in desiccators for drying. The spin-coated PES model surfaces were treated in the same way as described before using 20 mL 0.1 M sodium acetate buffer containing different concentrations of ferulic acid and laccase.

## 4. Conclusions

In this paper, the modification of PES membranes using ferulic acid modifier and the enzyme laccase was presented. The modified membranes showed a significant reduction in protein adsorption, while the clean water flux was not greatly reduced due to modification. Although, relatively dense modification layers of poly(ferulic acid) are formed, the clean water flux remains high. The obtained results of this study support the previous conclusions [17,27,28] in which there is no straight relationship between the amount of added material (GY) and total protein adsorption. In general, the addition of ferulic acid resulted in improvement in the static water contact angle on the model PES surface, which, consequently, increases the surface hydrophilicity. However, there is no direct relationship between the surface hydrophilicity and its protein repellence (*i.e.*, the modified model PES surface that shows the lowest contact angle does not correspond to the lowest total protein adsorption on the real membrane). Most probably, the internal polymer structure is the major effector on the created surface property (antifouling surface). SPM imaging assists the SEM and confirms the reported proposition that the modifier with three legs most probably grows as a pancake-like layer structure (in the three dimensions, 3D). Table 3 illustrates the difference between the ferulic acid modifier compared to two other modifiers (4-hydroxybenzoic acid and gallic acid) previously studied [28].

From Table 3, the GY of the ferulic acid is higher than the GY of the other two phenolic acids, whereas the layer thickness of the three modifiers is in the same range; perhaps, gallic acid produces the lower modifying layer thickness (model surfaces). Also, the flux reduction due to modification by ferulic acid is the highest among the three modifiers and this is due to large amount of material added. The reduction in the static water contact angle is better in case of 4-hydroxybenoic acid and ferulic acid than in the case of gallic acid. In general, the three modifiers are proposed to produce antifouling PES membranes, but a good modifier can be grown perpendicular on the membrane surface (to form brush-like oligomers/polymers). This is because good antifouling surfaces require both the steric hindrance of the added/grafted oligomers/polymers chains and the free polar groups to attract water and repulse the foulant molecules. 4-hydroxybenzoic acid and its similar structures are preferred for good foulant repellence. However, on the other hand, the addition/grafting of the both other modifiers (with more than two reactive legs) can be controlled to minimize the crosslinking and adsorption of the homopolymers formed inside the reaction medium. Moreover, ferulic acid showed good reduction in bacteria attachment as presented in previous work [33]. For that, the enzyme-catalyzed modification of PES membranes still needs further investigation to create novel antifouling or functionalized surfaces. For example, ferulic and 4-hydroxybenzoic acids can be used in combination, to collect the anti-biofouling properties of ferulic acid and the anti-protein fouling properties of 4-hydroxybenzoic acid. Also, further functionalization of the modification layer can be obtained by using modifiers that can subsequently react with other components, *i.e.,* with phenolic amines [39]. Finally, I am confident to say that the enzyme-catalyzed modification method is considered as the nucleus of a new generation of antifouling membranes using other modifier types and/or other membrane materials.

## Figures and Tables

**Table 1:** Analysis of XPS spectra of blank and modified real PES membranes. Modification condition is 28.8 mM ferulic acid, 24 h modification time, 0.1 M sodium acetate buffer (pH 5).

**Bindign Energy (eV)**	**C*_1s_***	**C*_1s_***	**O*_1s_***	**N*_1s_***	**S*_2p_***
	285.4 ± 0.3	288.8 ± 0.5	533.2 ± 0.3	400.1 ± 0.1	169.0 ± 0.3
	C–C	C=O	–C–O–	–N–	O=S=O
**Sample**	**Atomic %**				
Blank PES	77.01	16.99	15.56	2.15	5.300
Modified PES by Ferulic acid	74.77	9.985	22.38	1.01	1.845

**Table 2:** Flux reduction (% compared to blank) due to modification and (irreversible) protein adsorption under different modification conditions. Contact angle, layer thickness (model membrane), grafting yield, and total protein adsorption (reversible + irreversible) of some tested membranes in this study. Common modification condition is 4.8 mM ferulic acid, 0.5 U∙mL^−1^ laccase, 25 °C, pH 5, 0.1 M sodium acetate buffer, 2 h modification time.

Modification Condition	Flux Reduction % due to Modification ^a^	Flux Reduction% due to BSA Irreversible Adsorption ^b^	Total Flux Reduction% ^c^	Grafting Yield ^d^ (µg∙cm^−1^)	Total Protein Adsorption ^e^ (µg∙cm^−1^)	Contact Angle ^f^ (°)	Layer Thickness ^g^ (nm)
	Real Commercial Membrane					Model PES Surface	
Blank (Figure 5A and Figure 6A)	0	24.5	24.5	0	37.2	78.8	0
Common reaction (Figure 5B)	8.1	1.4	9.4	68.7	17.1	63.4	8.7
8 h Modification time (Figure 5C)	10.1	3.9	13.7	89.4	3.2	57.8	9.8
1 U∙mL^−1^ enzymeˑ (Figure 5F)	12.8	–	12.8	92.3	6.4	54.2	4.2
pH 7 (Figure 5I)	8.1	5.2	12.8	118	13.9	44.2	3.9
65 °C reaction temp.(Figure 5K)	8.1	–	8.1	76.4	2.2	64.4	13.4
Sodium citrate buffer (Figure 6C)	–	5.6	5.6	75.9	2.1	61.3	7.3

Typical errors: a = ±0.1, b = ±0.1, c = ±0.4, d = ±0.1, e = ±0.1, f =± 1, g = ±0.2.

**Table 3:** Comparison between the three modifiers: 4-hydroxybenzoic acid, gallic acid, and ferulic acid [17,27,28].

Parameter	4-hydroxybenzoic Acid	Gallic Acid	Ferulic Acid
Membrane color change	Membrane color change is a good indication for degree or extent of modification	Membrane color change is a good indication for degree or extent of modification	Membrane color change is NOT directly related to degree or extent of modification and color depth should be included (thick layers).
GY—average range	5–30 µg∙cm^−2^	5–30 µg∙cm^−2^	60–100 µg∙cm^−2^
Add material (%)	1–2.5	1–2.8	1–3.5
Flux reduction due to modification (%)	10–15	Max. 9	8–15
Layer thickness range (nm) (model surface)	3–15	2–10	3–16
Contact angle (°) (model surface)	63–51	70–62	67–44
Layer growing direction	Two dimensions (2D) brush-like structure	Three dimensions (3D) pancake-like structure	Three dimensions (3D) pancake-like structure
